# Factors Related to Oropharyngeal Dysphagia in Adults in a Healthcare Center in Colombia

**DOI:** 10.3390/healthcare13192421

**Published:** 2025-09-24

**Authors:** Lina Marcela Bernal Sandoval, Mónica Burgos García, Robinson Pacheco López

**Affiliations:** 1 Grupo de Investigación en Fonoaudiología y Psicologìa, Departamento Facultad de Salud, Universidad Santiago de Cali, Cali 760035, Colombia; monicaburgosgarcia@hotmail.com; 2 Facultad de Ciencias de la Salud, Universidad Libre Seccional Cali, Cali 760043, Colombia; robinson.pachecol@unilibre.edu.co

**Keywords:** dysphagia, risk factor, fluoroscopy

## Abstract

**Objectives**: We aimed to determine the frequency and factors related to oropharyngeal dysphagia in adults in a health center in Colombia evaluated by videofluroscopy of swallowing. **Methods**: We reviewed the records of 144 patients evaluated through videofluroscopy of swallowing. In order to analyze the results, descriptive, bivariate statistical analysis, and multivariate regression were used. **Results**: This investigation revealed that 23.6% of adults had oropharyngeal dysphagia. Older adults had a higher percentage of occurrence, and the factors associated with this symptom were having a history of cerebral stroke and being medicated with anticholinergic drugs. **Conclusions**: These findings strongly suggest that older adults with other comorbidities have a high percentage of presenting oropharyngeal dysphagia. Further research is needed to characterize the entity in other populations.

## 1. Introduction

During the swallowing process, alterations can occur that affect the correct pattern of bolus transit from the oral cavity to the stomach. This symptom is known as dysphagia, and indicates a loss of coordination, synchronism, and efficacy in food movement [1]. The swallowing process involves these in a complex and coordinated way, consisting of both voluntary and involuntary phases. The first phase is the oral phase, during which the tongue and masticatory muscles work together to form and propel the food bolus into the oropharynx. The second phase, known as the pharyngeal phase, involves structures such as the soft palate, epiglottis, and pharyngeal muscles, which help close the airway as the food bolus moves toward the esophagus. Finally, the esophageal phase occurs when the food is transported to the stomach through peristaltic movements of the esophagus, which are driven by smooth muscle and regulated by the lower esophageal sphincter [2]. The cortex primarily manages voluntary control of swallowing, while the bulbar centers reflex actions during the later stages. Additionally, the brain and central gray nuclei play a crucial role in the overall regulation of the swallowing process [3].

A swallowing disorder is diagnosed when dysphagia is confirmed to impair swallowing efficacy and safety. This includes both mechanical dysfunctions and subjective sensations related to swallowing. A definitive diagnosis requires a gold-standard assessment, such as a Videofluoroscopic Swallowing Study (VFSS) [4]. Dysphagia can occur at any age, and it is estimated that one in seventeen people may suffer from some swallowing disorder in their life [3]. In America, it is one of the ten most common causes of medical consultation, because it is a symptom that persists when faced with the perception of pain, discomfort, or difficulty in ingesting food [5]. These disorders can be associated with acute symptoms framed in colds, accompanied by gastroesophageal reflux disease, and even preceding sequelae of a non-transient disease.

In Colombia, some studies have estimated the prevalence of the perception of OD (oropharyngeal dysphagia) in 2023 in the country; a study from the SABE Colombia Survey showed a prevalence of 12.5% in a sample of 19,004 older people [6], but this survey is a self-report, so the respondent’s claim is not supported by objective examination. Some studies have shown that 35.5% of 31 people admitted to a Special Care Unit in Medellin have dysphagia. The most prevalent diagnoses in this population were head and neck cancer and cardiovascular diseases [7]. In Bogota, a study conducted in a specialized unit for esophageal cancer management found that 64.28% of 36 patients had a swallowing disorder [8]. This shows that this disorder has multiple causes and its frequency varies. However, the overall frequency of swallowing disorders has not been determined [7]. In general, the samples to analyze this behavior are small and the results cannot be generalized.

The factors related to anatomical changes due to increasing age, and neurodegenerative and cerebrovascular diseases can significantly increase the risk, affecting the processing and oral containment necessary for bolus propulsion and impacting safety due to the appearance of false routes in the transit of the food bolus or accumulation in the pharyngeal tract [5]. As a result, dysphagia compromises the nutritional capacity and the indemnity of the respiratory system [6]. Aspiration of food, oral secretions, or gastroesophageal material can lead to aspiration pneumonia, a condition recognized by the International Classification of Diseases (ICD-10) [9]. This condition generally requires intensive care because it causes severe lung damage that develops rapidly, accompanied by acute respiratory distress syndrome and a series of systemic complications that require special attention [10]. This situation increases healthcare costs, limits patient recovery, and substantially worsens quality of life.

However, the interdisciplinary teams of healthcare institutions must identify the factors that contribute to the appearance of the condition. This generates the ability to prioritize the examination and the generation of formal evaluation and treatment plans for the safe provision of foods, fluids, and oral medications [11]. For this reason, recognizing, measuring, and characterizing the related factors of a diverse group of people of different ages and pathologies provides relevant information on the frequency and possible associations that determine the epidemiological behavior of this entity. The lack of concrete data on the frequency of OD in Colombia represents a major knowledge gap, limiting our comprehensive understanding of its clinical, social, and economic burden. Without robust local information, it is difficult to accurately measure its prevalence, incidence, and associated factors. This makes it challenging to identify at-risk populations and prioritize effective interventions.

The Colombian Health System, through the Health Benefits Plan (PBS—Plan de Beneficios en Salud), offers access to radiological examinations financed by Capitation Payment (UPC), an annual monetary allocation that the General System of Social Security in Health (SGSSS—Sistema General de Seguridad Social en Salud) allocates to each affiliate [12]. However, VFSS is not explicitly detailed within the PBS; therefore, health professionals must request authorization through a Technical and Scientific Committee (CTC) or its equivalent, justifying the clinical need for the exam. This process can cause considerable delays in approval. In addition, the availability of this technology varies by region, as significant inequalities persist between urban and rural areas, as well as between the different Benefit Plan Administration Entities (EAPB—Entidades Administradoras de Planes de Beneficios) and their capacity to offer appointments and specialized services.

This study aims to determine the frequency of oropharyngeal dysphagia and analyze its associated factors in adults evaluated at a single healthcare facility in Cali, Colombia. We focused on identifying key risk factors and their independent associations with OD.

## 2. Materials and Methods

A cross-sectional observational study was conducted, in which VFSS was applied to 190 patients in a Health Services Provider Institution in Santiago de Cali, Colombia, from 2018 to 2020. The sampling was non-probabilistic at convenience because all records of such procedures were included.

We considered the following inclusion criteria: (1) patients older than 18 years with suspected occasional or frequent symptoms who were referred by their treating physician as supporting evidence for the referred symptomatology; (2) those patients who reported at least one baseline disease in their medical history. The exclusion criteria we considered were (1) follow-up exams of previously diagnosed patients OD, and an (2) incomplete procedure.

To ensure consistency in the application of the protocol, a standardization process was carried out among the evaluators. Two of the three investigators involved in data collection participated in theoretical and practical training sessions, during which diagnostic criteria, assessment techniques, and the use of study instruments were reviewed in detail. Also, joint evaluation exercises using simulated cases were conducted to unify criteria and minimize variation in the interpretation of findings. As part of quality control, inter-rater reliability measures were calculated and Cohen’s kappa coefficient was used for categorical variables, and the intraclass correlation coefficient (ICC) was applied for continuous variables. The resulting values were within acceptable ranges, indicating good agreement.

The analysis of the examination is referenced under the minimum conditions inscribed in the manual of good practices for the performance of swallowing tests with fluoroscopy (application in contrast images) for the oral, pharyngeal, and esophageal domains. The components of the swallowing mechanism that were analyzed were lip closure, lingual control of the bolus, preparation, bolus transport, oral residue, swallow reflex, soft palate elevation, anterior hiatus excursion, epiglottic movement, laryngeal vestibule closure, posterior pharyngeal constriction, the opening of the pharyngeal esophageal transition, pharyngeal residue, and esophageal emptying.

Patients were classified as having oropharyngeal dysphagia based on objective findings obtained through a VFSS. The diagnosis was made according to internationally recognized criteria, which included signs such as delayed swallow initiation, pharyngeal residue, penetration or aspiration (as defined by the Penetration–Aspiration Scale, PASS [13], and the DOSS scale [14]).

Although the severity of dysphagia was initially assessed using those standardized scales, the final analysis presented the presence or absence of dysphagia as a dichotomous variable (“Yes” or “No”). This decision was made to facilitate statistical analysis and to focus on identifying factors associated with the occurrence of dysphagia rather than its gradation. Dichotomization was performed based on predefined cutoff points on each scale. For the PAS, scores of ≥3 (indicating penetration or aspiration) were considered indicative of dysphagia. For the DOSS, scores of ≤5 (reflecting mild to severe dysphagia) were categorized as positive for dysphagia. Patients scoring below these thresholds were classified as “No” for dysphagia.

While this approach may reduce the granularity of clinical interpretation, it allowed for a clearer comparison of prevalence and associated risk factors. The original severity scores were retained for internal reference and may be explored in greater depth in future analyses.

These results were consolidated in a database in Microsoft^®^ Excel 2016 by the researchers. Within the database, the variables of sex, age, ethnicity, diseases, type of medication used, and the presence or absence of oropharyngeal dysphagia were established. To maintain patient confidentiality and ensure recognition within the study, names were omitted, a numerical coding system was implemented, and the records were only analyzed directly in the teams of the Health Institute; this approach was intended to ensure greater accuracy and completeness of the information, minimizing the risk of recall bias or documentation errors typically associated with retrospective data abstraction from medical records.

The methodology also included a new review of the collected data to identify any missing values. In cases where missing data were encountered, they were explicitly documented. No data imputation methods were applied, as the proportion of missing data was minimal and did not significantly affect the statistical power of the study; therefore, they were excluded from the analysis.

Stata12^®^ software (StataCorp, College Station, TX, USA) was used for statistical analysis. Descriptive statistics were used for the univariate analysis of the clinical and demographic characteristics, the means were calculated with their respective standard deviation, and the nominal variables were summarized using proportions. Utilizing a bivariate analysis between those who did and did not present OD, an odds ratio (OR) was used, looking for differences with their respective 95% confidence intervals (CI). A *p*-value of less than 0.05 was considered statistically significant. Finally, multivariate logistic regression was performed to estimate the OR considering the variables of interest in the bivariate analysis. The multivariate model was built by backward elimination with *p* < 0.20 in the initial iterations to retain possible confounders, requiring *p* < 0.05 in the final model.

## 3. Results

Between 2018 and 2020, 190 adults were evaluated by swallowing objective test. A total of 46 results (24.2%) were suppressed due to the criteria established for the study’s execution. Finally, 144 data were analyzed. Of these, 23.61% (34/144) were diagnosed with oropharyngeal dysphagia, and 76.39% (110/144) had a negative diagnosis for this event.

The median age was 70 years. Of those included, 61.1% (88/144) were women, 77.08% were mixed race (111/144), 47.92% (69/144) had cardiovascular diseases, and 29.86% (43/144) had cerebrovascular diseases that were more frequent. All the clinical and sociodemographic characteristics are listed in Table 1.

During the bivariate analysis, people with cerebrovascular disease had a significantly higher probability of presenting OD (OR = 6.46, CI 95%: 2.59–16.26; *p* < 0.001). In contrast, people with a medical history of gastrointestinal disease had a lower probability of having OD (OR = 0.11, CI 95%: 0.02–0.77; *p* < 0.01). Table 2 shows the bivariate analysis.

From the initial bivariate analysis, nine variables with *p* < 0.20 were included in the saturated logistic regression model. Through backward elimination, we identified two independent variables that explain the factors related to the occurrence of swallowing disorders in the population, which are records of patients with a history of cerebrovascular disease (aOR = 8.96, CI 95%: 3.55–22.64.; *p* < 0.000) and the use of anticholinergic drugs as pharmacological treatment (aOR = 1.69, CI 95%:1.50–16.17; *p* < 0.008). Table 3 shows the logistic regression.

## 4. Discussion

The present study aimed to determine the frequency of dysphagia in older adults in people assessed using Videofluoroscopic Swallowing Studies (VFSSs), and to identify associated factors. Dysphagia is a significant health concern as it directly affects the well-being of individuals and leads to increased costs in the healthcare system due to associated comorbidities [5]. It is important to recognize this entity within a specific setting such as the one studied. Different authors have affirmed that the existing evidence related to its etiology and epidemiological behavior is varied and is expressed in multiple factors related to the context [15].

The considerations analyzed here emphasize that swallowing disorders occur more in older adults, which could be related to the presence of presbyphagia because the aging process is related to neurophysiological and anatomical changes that affect the sensory and neuromotor response of the isometric response to perform the swallowing process [9]. However, from a differentiating point of view between oropharyngeal dysphagia and presbyphagia [16], it is important to mention that the differences are centered on the consequences of pathological, neurological, muscular, mechanical, or extrinsic affectations such as surgery or cancer treatments. At the same time, old swallowing, typical of age, does not affect swallowing safety. Therefore, it would be necessary to consider whether most of the patients that a treating physician sent for evaluation in this study were sent regarding a geriatric syndrome, in agreement with [17].

Cancer is a highly heterogeneous condition that is associated with dysphagia not only due to the anatomical location of the primary tumor but also because of the sequelae of oncological treatments, including surgery, radiotherapy, and/or chemotherapy. These therapeutic interventions can compromise the structural and functional integrity of the upper aerodigestive tract, affecting both the safety of swallowing and the efficiency of bolus transit, which directly impacts the patient’s ability to maintain adequate and complete oral intake. Therefore, it is essential that future studies consider these clinical variables to better understand the specific impact of cancer and its treatments on swallowing function [17]; although, in our study, this condition was not statistically significant, the literature shows a strong relationship.

One of the diseases related to OD that is a factor in this study is cerebrovascular disease. In Colombia, it is among the five principal comorbidities, causing 32 deaths per 100,000 inhabitants [18]. This condition affects the cortical areas responsible for swallowing and may impact both the pharyngeal and oral phases, depending on the location of the lesion, as altered neurological conditions can lead to a decreased ability for individuals to feed themselves independently [19], particularly in regions such as the brainstem and motor cortex, leading to impaired coordination and strength of the muscles involved in swallowing. This disruption results in delayed swallow initiation, reduced pharyngeal contraction, and impaired airway protection, all of which contribute to dysphagia [20]. Changes in dietary intake, modifications to food consistencies, and reliance on enteral nutrition can significantly affect the nutritional status of these patients.

In our sample, digestive conditions did not play a significant role in the development of swallowing difficulties during the oropharyngeal phase. People with those kind of problems often describe sensations like food getting stuck, chest discomfort, or regurgitation, and without a proper diagnostic evaluation, these symptoms can easily be mistaken for difficulties in the initial phase of swallowing, leading to confusion in both self-reporting and clinical assessment because, while oropharyngeal dysphagia is usually related to neurological or structural problems in the head and neck, gastrointestinal diseases more commonly affect the esophageal stage of swallowing [21].

It is also important to mention that 55.5% of the individuals in this study were medicated with antihypertensive and although they did not show significance related to OD, it is important to highlight that angiotensin-converting enzyme inhibitors (ACE inhibitors) reduce the response of the sympathetic nervous system [22]. This drug can cause a dry cough and gastroesophageal reflux, as well as direct signs of a reduced and modified salivary excretory capacity. As a result, the perception of food bolus transit may be altered, and some swallowing disturbances may be confounded [23]. Therefore, an objective instrumental test should be applied to avoid false positives. In the Colombian context, access to Fiberoptic Endoscopic Evaluation of Swallowing (FEES) is limited due to various factors, including its low utilization among clinicians and its exclusion from the official list of procedures covered by the national healthcare system. These constraints significantly reduce its availability in clinical practice. In contrast, Videofluoroscopic Swallowing Study (VFSS) remains the most accessible and widely used diagnostic tool in the country, serving as the primary instrumental method for the objective assessment of dysphagia.

The drugs that finally did show an association were those with anticholinergic action, and they were identified similarly in other studies [24,25]. Anticholinergic medications contribute to dysphagia primarily through their inhibition of acetylcholine, a neurotransmitter that is essential for parasympathetic nervous system activity. By blocking muscarinic receptors, these drugs reduce salivary secretion, leading to xerostomia (dry mouth), which impairs bolus formation and lubrication during swallowing. Additionally, anticholinergic effects can cause decreased smooth muscle contractility in the esophagus and pharynx, further disrupting the swallowing process [26]. These findings highlight the need to study dysphagia related to this drug, as an adverse event modifying the health and integrity of the patient, and to highlight it as a warning in the nutritional aspect and respiratory indemnity because of the risk of developing aspiration pneumonia [27]. A systematic review, complemented by real-world data analysis from the FDA Adverse Event Reporting System (FAERS), evaluated the effects of angiotensin-converting enzyme inhibitors (ACEis), beta-blockers, and dipeptidyl peptidase-4 inhibitors (DPP-4i) in patients with neurological disorders presenting with dysphagia and an increased risk of aspiration pneumonia. The findings suggest a potential therapeutic effect mediated by the enhancement of the pharyngeal reflex, attributed to elevated levels of substance P and modulation of the central and peripheral nervous systems involved in swallowing control. These results underscore the need for further clinical research to validate and better understand the underlying mechanisms [28].

From an epidemiological perspective, it is important to understand that the estimation of the global prevalence of OD is limiting due to the geographic variability in the burden of disease, influenced by sociocultural and biological determinants. So, this highlights the need for regionally stratified studies.

This study has limitations that should be mentioned. It is necessary to expand the population sample in future studies to obtain more representative results; this study was limited by the sample size, which could introduce biases and reduce the generalizability of the findings. Incorporating greater diversity in terms of age, gender, and medical history allows for the identification of other associations. In addition, a larger sample will provide a more robust statistical basis, increasing the reliability and validity of the conclusions obtained; the estimates obtained from this sample presented a wide CI, which could vary if the study is repeated in another population, affecting the reliability of the conclusions; multicenter studies could be chosen to estimate the magnitude of the event from a more robust sample. The non-probabilistic convenience sampling in our study was not representative of the target population because participants were chosen based on their accessibility or availability at the health center, incurring probable systematic errors and affecting a real estimate.

Our study represents an initial descriptive phase designed to identify factors associated with oropharyngeal dysphagia in our target population. While this cross-sectional design did not allow an evaluation of clinical outcomes such as nutritional complications, aspiration pneumonia, or quality of life impact, the obtained results provide the necessary epidemiological foundation to justify and design subsequent longitudinal studies.

Therefore, conducting a multicenter study with probability sampling would be the most effective approach. The conduct of this study was immersed with the beginnings of COVID-19, which meant that restrictive measures were implemented to administer tests and patient capacity was limited due to lower flow. In addition, the OD severity scale and other associated events such as the history of invasive mechanical ventilation were not considered. Future studies should focus on conducting prospective, multicenter investigations with larger and more diverse sample sizes to enhance the generalizability of findings. It is also important to explore additional factors that may influence oropharyngeal dysphagia, including dietary habits, levels of physical activity, and the use of other classes of medications. Moreover, longitudinal studies could provide valuable insights into the causal relationships and progression of dysphagia over time, and while the multivariate model accounts for error accumulation, the bivariate findings should be interpreted with caution and considered exploratory to inform future research.

## 5. Conclusions

Our findings show that nearly one in four adults in our study experienced oropharyngeal dysphagia, with older adults being the most affected. We found that people with a history of cerebrovascular disease, as well as those taking medications with anticholinergic effects, were significantly more likely to have swallowing difficulties. It was also determined that not all people who are evaluated by VFSS have a positive diagnosis of dysphagia. It is important to continue research on this area, characterizing another type of population and optimizing the diagnostic resource as well as the test. This study provides a valuable contribution by identifying the prevalence of oropharyngeal dysphagia and its significant associations with cerebrovascular disease and anticholinergic medication use in adults attending a healthcare center in Colombia. While acknowledging the exploratory nature and limitations of the study, these findings highlight the importance of early identification and management of dysphagia in at-risk populations. Further research, as outlined, is essential to deepen understanding and improve clinical outcomes.

## Figures and Tables

**Table 1:** Socio-demographic and clinical characteristics of the population.

Characteristics Socio-Demographic	Description	Measure
Age	Years	Median 71.5 [IQR 37–96] *
Sex (%)	Men (n = 56)	56 (38.8)
	Women (n = 88)	88 (61.11)
Ethnicity (%)	Mixed race (n = 111)	111 (77.08)
	Afrodescendent (n = 33)	33 (22.91)
Clinical characteristics	Description	Measure
Diseases (%)	Cancer	16 (11.11)
	Endocrinology	32 (22.22)
	Cardiovascular	69 (47.92)
	Cerebrovascular	43 (29.86)
	Mental	42 (29.17)
	Respiratory	21 (14.58)
	Gastrointestinal	24 (16.67)
Medication	Antihypertensives	80 (55.56)
	Hormonal	39 (27.08)
	Non-steroidal anti-inflammatory drugs (NSAIDs)	32 (22.22)
	Antidepressants	31 (21.68)
	Antipsychotics	25 (17.68)
	Anticholinergics	18 (12.50)

* IQR: Interquartile range.

**Table 2:** Risk factors related to swallowing disorders, as found in bivariate analysis.

Characteristics	Description	Dysphagia	Non-Dysphagia	OR *	CI95% **	*p-*Value
Age	Median	75.5 [IQR 64–88]	71.5 [IQR 56–81]	1.02	0.99–1.04	0.144
Sex	Men	18	70	0.34	0.14–0.80	0.064
	Women	16	40			
Ethnicity	Mixed race	27	84	0.14	0.27–0.83	<0.007
	Afrodescendent	7	26			
Cancer	Yes	5	11	1.55	0.38–5.32	0.44
	No	29	99			
Endocrinology	Yes	5	27	0.53	0.14–1.58	0.22
	No	29	83			
Cardiovascular	Yes	16	53	0.95	0.40–2.21	0.90
	No	18	57			
Cerebrovascular	Yes	21	22	6.46	2.59–16.25	<0.00
	No	13	88			
Mental	Yes	10	29	1.72	0.69–4.16	0.18
	No	24	81			
Respiratory	Yes	3	18	0.49	0.08–1.87	0.27
	No	31	92			
Gastrointestinal	Yes	1	23	0.11	0.02–0.77	<0.01
	No	33	87			
Hormonal medications	Yes	6	33	0.5	0.15–1.39	0.15
	No	28	77			
Antidepressant medications	Yes	7	24	0.91	0.30–2.52	0.85
	No	27	85			
Antihypertensive medications	Yes	21	59	1.39	0.59–3.35	0.40
	No	13	51			
Anticholinergic medications	Yes	11	7	2.33	0.69–7.31	0.10
	No	27	99			
NSAIDs	Yes	11	21	2.02	0.76–5.15	0.10
	No	23	89			
Antipsychotic medications	Yes	9	16	2.21	0.72–5.79	0.10
	No	25	94			

* OR: odd ratio; ** CI: confidence interval.

**Table 3:** Logistic regression of factors related to oropharyngeal dysphagia, found via multivariate analysis.

Characteristics	Description	Total	OR	aOR *	*p-*Value
Cerebrovascular disease	Yes	43	6.46	8.96	<0.000
	No	101	CI95% (2.59–16.25)	CI95% (3.55–22.64)	
Anticholinergic medications	Yes	18	1.71	1.69	<0.008
	No	126	CI95% (0.69–7.31)	CI95% (1.50–16.17)	

* Adjusted odds ratio.

## Data Availability

The data are not publicly available and are subject to a use agreement between the researchers and the health clinic.
